# Fatigue in Multiple Sclerosis: General and Perceived Fatigue Does Not Depend on Corticospinal Tract Dysfunction

**DOI:** 10.3389/fneur.2019.00339

**Published:** 2019-04-09

**Authors:** Laura Mordillo-Mateos, Vanesa Soto-Leon, Marta Torres-Pareja, Diego Peinado-Palomino, Nuria Mendoza-Laiz, Carlos Alonso-Bonilla, Michele Dileone, Mario Rotondi, Juan Aguilar, Antonio Oliviero

**Affiliations:** ^1^FENNSI Group, Hospital Nacional de Parapléjicos, Servicio de Salud de Castilla La Mancha, Toledo, Spain; ^2^Facultad de Ciencias del Deporte, University of Castilla La Mancha, Toledo, Spain; ^3^CAFyD, Universidad Francisco de Vitoria, Madrid, Spain; ^4^Advanced Neurorehabilitation Unit, Hospital Los Madroños, Madrid, Spain; ^5^Unit of Internal Medicine and Endocrinology, Laboratory for Endocrine Disruptors, IRCCS Scientific Clinical Institutes Maugeri, University of Pavia, Pavia, Italy; ^6^Experimental Neurophysiology, Hospital Nacional de Parapléjicos, Servicio de Salud de Castilla La Mancha, Toledo, Spain

**Keywords:** fatigue, motor evoked potential, compound motor action potential, multiple sclerosis, motor cortex

## Abstract

**Background:** Multiple sclerosis (MS) is an autoimmune disorder of the CNS in which inflammation, demyelination, and axonal damage of the central nervous system coexist. Fatigue is one of the most disabling symptoms in MS and little is known about the neurophysiological mechanisms involved.

**Methods:** To give more mechanistic insight of fatigue in MS, we studied a cohort of 17 MS patients and a group of 16 age-matched healthy controls. Baseline Fatigue Severity Scales and Fatigue Rating were obtained from both groups to check the level of fatigue and to perform statistical correlations with fatigue-induced neurophysiologic changes. To induce fatigue we used a handgrip task. During the fatiguing task, we evaluated fatigue state (using a dynamometer) and after the task we evaluated the Borg Rating of Perceived Exertion Scale. Transcranial magnetic stimulation and peripheral electric stimulation were used to assess corticospinal tract and peripheral system functions before and after the task.

**Results:** Clinically significant fatigue and central motor conduction time were greater in patients than in controls, while motor cortex excitability was decreased and maximal handgrip strength reduced in patients. Interestingly, fatigue state was positively correlated to perceived fatigue in controls but not in patients. Furthermore, in the presence of similar fatigue state over time, controls showed a significant fatigue-related reduction in motor evoked potential (a putative marker of central fatigue) whereas this effect was not seen in patients.

**Conclusions:** in MS patients the pathogenesis of fatigue seems not driven by the mechanisms directly related to corticospinal function (that characterize fatigue in controls) but seems probably due to other “central abnormalities” upstream to primary motor cortex.

## Introduction

Multiple sclerosis (MS) is an immuno-mediated disorder of the central nervous system (CNS) in which inflammation, demyelination, and axonal damage coexist (1). MS prevalence and incidence have increased progressively over time (2). Importantly, MS is commonly diagnosed between 20 and 40 years of age, affecting the early stages of working lives and leading to a further increase in social costs (3, 4). MS is characterized by sensory and motor symptoms, bladder and bowel problems, cognitive impairment and fatigue. Fatigue trait in MS was defined, by the Multiple sclerosis council for clinical practice guidelines, as “a subjective lack of physical and/or mental energy that is perceived by the individual or caregiver to interfere with usual and desired activities”(5). It occurs frequently and is considered one of the most disabling symptom since it interferes with the performance of daily activities (6). However, the pathophysiology of fatigue and the mechanisms involved are still poorly understood (7–13).

On the other hand, fatigue state, can be defined as the decline in peak force (torque) after performing an exercise intervention (14). Generally, fatigue triggered by overstrain in healthy subjects, is mediated by muscular factors even if changes in the peripheral and central nervous system as well as lack of self-motivation (15) or a subjective decrease in mental/physical energy (16) are also involved (7, 17–19).

In this line, in healthy subjects it was demonstrated that the development of fatigue depends on the changes affecting several structures such as spinal cord, cerebral cortex and subcortical structures (17, 20, 21). Accordingly, it was recently shown that fatigue in MS patients depends on the changes of functionality of CNS (10, 11, 21–26)]. Since the impact of fatigue in daily life of MS patients is high and since 80–90% of MS patients present with fatigue (26), a more profound knowledge of the mechanisms involved in the fatigue development could help to better evaluate patients and ultimately could help to find new treatments.

Although several works seem to suggest that MS fatigue is associated to corticospinal dysfunction, other works did not confirm this causal link (10, 21, 23). Moreover, in healthy subjects not-fatiguing tasks did not lead to reduction in Motor Evoked Potentials (MEP) (i.e., post exercise depression) (27), while studies evaluating post-exercise depression of MEP amplitude in MS patients demonstrated variable results (increased in one study, reduced in another and similar to healthy subjects in 3 studies) and that the amount of post-exercise depression was not related to self-reported fatigue in MS (28).

In the present study, we aimed to give more mechanistic insights about the lack of post exercise depression in fatigued MS patients, by studying the effects of a fatiguing hand task on motor cortex and peripheral nerve functions and by looking for any correlation between neurophysiological parameters of central and peripheral fatigue, maximal handgrip strength (MHS) and subjective perception of fatigue. We hypothesized that the lack of post exercise depression in fatigued MS patients could be explained by different neurophysiological alterations in motor cortex and peripheral nervous system functions.

## Methods

### Participants

We enrolled 17 consecutive MS patients (4 males) and 16 healthy controls (8 males). The study protocol was approved by the Ethical Committee for Clinical Research (Toledo) and was performed according to the Declaration of Helsinki. All participants gave written informed consent prior to participation. Patients and controls were asked to quantify their weekly physical activity referring to recreational activity: they were asked for how many days a week and for how many hours a day they perform vigorous recreational activity (such as sport, fitness and other aerobic sports). Subjects that did not perform at least 3 h /week did not enter the study. All controls and patients were right-handed: hand's dominance was checked by means of an interview (i.e., we asked the patients and subjects about their dominance).

Healthy subjects had a mean age (± SD) of 33.2 ± 12.4 years (range: 24–59 years), with no neurologic or psychiatric diseases.

Patients were diagnosed with clinically definite MS (24) and had sufficient upper limb motor function to perform the handgrip task [Medical Research Council (MRC) muscle scale (rated from 0 to 5) in flexor and extensor hand muscles > 3]. MS patients (mean age ± SD: 36.3 ± 9.5 years; range: 29–59 years; controls vs. patients: *p* = 0.42) were enrolled from the ADEMPTO (The Multiple Sclerosis Patients' Association of Toledo, Spain). No significant differences were found for male/female ratio (*p* = 0.114). Expanded Disability Status Scale (EDSS) were used to evaluate neurological impairment and disability of the MS patients (22). No patients had clinical relapse in the 3 months preceding the study. All patients had clinical evidence of pyramidal signs (i.e., hyposthenia, augmented tendon reflexes, Babinski sign, etc.). Inclusion criteria were: (a) definite MS diagnosis; (b) disease duration > 3 years; (c) no cognitive impairment or any substantial decrease in alertness, language reception, or attention that might interfere with understanding instructions for motor testing; (d) no known peripheral nerve pathologies affecting upper limb; (e) no concomitant neurological conditions, including any history of epilepsy and significant comorbidities; (f) no apraxia; (g) no excessive pain in any joint of the arms; (h) no contraindications to TMS such as metal head implants or cardiac pace-makers; (i) no advanced liver, kidney, cardiac or pulmonary disease; (j) no history of significant alcohol or drug abuse; (k) no major depression or severe psychiatric disorder; (l) practicing physical activity at least 3 h/week.

Clinical and demographic data of MS patients are described in Table 1.

**Table 1:** Clinical and demographic data of MS patients.

**Subject**	**Clinical course**	**Disease duration (years)**	**EDSS**	**Pharmacological treatment**				
				**DMD**	**Spasticity**	**Pain**	**Fatigue**	**Others**
1	SP	25	7		Baclofen		Fampidrine	
2	RR	15	1					
3	SP	24	6.5	Azathioprine	Cannabidiol		Modafinil	
4	RR	20	4.5	Interferon beta-1a				Tolterodine Tartrate, Escitalopram
5	RR	19	3.5	Dimethyl- fumarate	Clonazepam			Venlafaxine
6	SP	20	6	Interferon beta-1a	Baclofen		Amantadine	
7	SP	15	6	Fingolimod	Cannabidiol			Fluoxetine
8	SP	22	8		Baclofen			
9	SP	21	6.5		Clonazepam		Amantadine Fampidrine	
10	RR	9	2	Dimethylfumarate				
11	RR	27	5.5	Interferon beta-1a			Fampidrine,	Escitalopram
12	RR	5	5.5	Rituximab		Pregabalin		ASA, Simvastatin,Trazodone
13	RR	8	4.5					
14	SP	5	5			Gabapentin		Latanoprost, Melatonin
15	RR	3	2	Interferon beta-1a				
16	RR	6.5	6.5	Interferon beta-1a	Baclofen			
17	SP	6	6	Azathioprine	Baclofen, Cannabidiol	Pregabalin		Omeprazol

*EDSS, Expanded Disability Status Scale; DMD, disease modifying drugs; SP, Secondary Progressive; RR, Relapsing Remitting*.

All participants, after signing the informed consent, were interviewed and clinically evaluated by a neurologist to check for no gross signs of dementia, psychiatric disorders and/or apraxia and deficit of attention and completed the Fatigue Severity Scale (FSS). FSS is a self-administered 9-items-questionnaire that measures the severity of habitual fatigue in different situations with each item ranging from 1 to 7 (1 indicates strong disagreement and 7 strong agreement), and with the final score representing the mean value of the 9 items. Clinically significant fatigue (CSF) was defined as FSS scores greater than or equal to 4 (29).

Moreover, participants were asked to rate fatigue (fatigue rating – FR) from 0 (no fatigue) to 10 (extreme fatigue) by means of a visual analog scale.

## Motor Task

To study fatigue state, we used a handgrip task of 2 min in which subjects were asked to squeeze a hand dynamometer to produce maximal voluntary contraction: subjects were asked to maintain their maximal strength during the whole duration of the task and were verbally encouraged to provide maximum contractions. We a priori decided to evaluate the left hand in control group while both hands, in two separated sessions, were evaluated in MS patients.

Handgrip task was performed by using a hand-held dynamometer (Cibertec SA, Spain) while the subjects were seated with their shoulder adducted and neutrally rotated, elbow flexed at 90°, forearm in neutral position, and wrist between 0 and 30° dorsiflexion and between 0 and 15° ulnar deviation. During grasping, subjects were instructed to flex their fingers. Data were recorded on computer for later analysis using a CED 1401 A/D converter and Spike software (Cambridge Electronic Design, Cambridge, UK). Maximal handgrip strength (MHS) was defined as the highest peak torque (Newton, N) obtained during the 2 min handgrip task for each participant. The entire dynamometer signal (2 min) was analyzed by dividing it in 20 s' blocks (6 blocks in total, namely B1-B6). For each block, the modulus of the handgrip strength (HS) was quantified and data were expressed as Newton per seconds (N^*^s). Force decay (i.e., modulus decay; FD) over the 2 min was considered as a marker of fatigue trait (last 20 s/first 20 s^*^100). Modulus is defined as area over the zero baseline but with all absolute values.

## Evaluation of Perceived Fatigue

At the end of each session, all subjects were asked to report their level of perceived intensity during handgrip using the Borg Rating of Perceived Exertion Scale (BRPES, range 6–20, where 6 means no exertion at all and 20 means maximal exertion). Although this is a subjective measure, a person's exertion rating may provide a fairly good estimate of physical activity (30). The total HS modulus of the whole task (THS- N^*^s) was also calculated to quantify the total effort of each subject/patient. The ratio between the whole task modulus (root squared to reduce variability) and BRPES were also calculated. This indicates the effort perception relative to the continuous motor activity.

## Neurophysiological Study

We aimed to study the effects of an isometric handgrip-fatiguing task on peripheral and central motor excitability of controls and MS participants (case-control study design).

For this reason we used Transcranial Magnetic Stimulation (TMS) to evaluate central motor excitability and peripheral nerve stimulation for peripheral motor excitability.

TMS was performed to evaluate central motor excitability by assessing MEP modulus and central motor conduction time (CMCT) (see below for details) of both upper limbs in MS patients and of left upper limb in controls; these parameters were used as putative markers of corticospinal integrity (31).

Peripheral nerve electrical stimulation (pES) of the ulnar nerve was used to evaluate peripheral motor excitability by assessing compound muscle action potentials (CMAP) of the first dorsal interosseous (FDI) muscle (amplitude and latency) and the F wave latency (see below for details), again for both hands in MS patients and for left hand in controls.

Central motor excitability and peripheral nerve excitability were evaluated before and after a 2-min handgrip task both in MS patients and healthy controls.

### Transcranial Magnetic Stimulation

TMS recordings were performed while subjects at rest, with arm relaxed, elbow flexed at 90 degrees with the forearm and supinated hand lying on an armrest. Focal TMS of the hand area of right primary motor cortex (M1) was performed with a high-power Magstim 200 (Magstim Co., Whitland, UK), which delivers monophasic pulses. A figure-of-eight coil (model D70mm Alpha, Magstim Co., Whitland, UK) was held over the optimum scalp position to elicit motor responses in the FDI. Intensities were expressed as a percentage of the maximum output of the stimulator. Throughout all the experiments, TMS was applied with the handle of the coil pointing backwards, with the induced current flowing in a posterior-anterior (PA) direction. The optimum coil position was defined as the site where TMS consistently resulted in the largest MEP. Surface electromyography (EMG) was recorded from the FDI using adhesive electrodes in a belly-tendon montage. EMG signals were band-pass filtered (3Hz−3kHz) (Digitimer D360 amplifiers) and single trials were digitized (sampling rate 10kHz) and recorded on computer for later analysis using a CED 1401 A/D converter and Spike software (Cambridge Electronic Design, Cambridge, UK).

Resting motor threshold (RMT) was defined as the minimum stimulus intensity that produced a liminal MEP (>50 μV in 50% of 10 trials) with the tested muscle at rest (32, 33). MEPs were obtained by recording the responses from the relaxed FDI to 20 single-pulse TMS stimuli (5 s inter-stimulus interval).

Both for patients and controls, MEPs were obtained by recording the responses from the relaxed FDI to 20 single-pulse TMS and 20 peripheral nerve stimuli (5 s inter-stimulus interval). TMS intensity was set at 120% RMT and the same intensity was used at all-time points after fatiguing task. Modulus of MEPs (MEP_mod_) were off-line calculated (mV^*^sec) and utilized as the value of the dependent variable. Those that exceeded 2 standard deviations from the session average or that were preceded by clear EMG activation were manually rejected. MEP_mod_ decrement after fatiguing task at T1 was also calculated as percentage of baseline value (T1/T0^*^100). We will refer to this variable as motor system fatigability (MF): the smaller the resulted value the bigger the MF.

Minimal MEP latency was measured to calculate CMCT (see below).

### Peripheral Electrical Stimulation

The ulnar nerve was stimulated via electrodes placed ~4 cm apart along the ulnar side of the wrist. Stimuli were delivered to the electrodes from a constant current stimulator (pulse width 200 μs, DS7A; Digitimer, UK) controlled by Spike-2 7.0 software (Cambridge Electronic Design Limited). EMG signals were obtained with the same parameters than used for TMS and from the same muscle (FDI).

Electrical RMT (eRMT) was defined as the minimum stimulus current required to evoke a CMAP of amplitude of approximately 50 μV and this parameter was used to set stimulation intensity for the experimental session. Then, stimulation intensity was set at 150% eRMT for peripheral stimulation (34) and then the same intensity was used at all-time points after the fatiguing task.

Modulus of the CMAPs were off-line calculated (mV^*^s) and utilized as the value of the dependent variable.

Furthermore, only in baseline condition, 10 supramaximal stimuli were delivered to obtain an F wave (and M wave) to allow CMCT calculation (see below). To obtain supramaximal stimulation, the stimulus intensity was increased in 5–10 mA to a level 10% higher than the point where the resultant CMAP did not increased in modulus.

### Central Motor Conduction Time

MEP latency (ms) provides evidence of the integrity of cortico-spinal tract; particularly the latency of the MEP reflects the conduction time for neural impulses from the cortex to muscles (35) and was determined as the minimum time recorded between TMS shock and MEP onset during the entire baseline recording block. MEP latency was used to calculate the CMCT by subtracting the peripheral conduction time (PCT) to the MEP latency. Peripheral conduction time was calculated using F wave approach and the following formula: (minimal F wave latency + M wave latency-1)/2 (36).

### Experimental Design: Effects of Induced Fatigue on Central and Peripheral Nervous System

Only in baseline condition we evaluated CMCT, F wave and M wave to supramaximal stimuli (bilaterally in patients and in the left hand in controls) for neurophysiological measures and FSS and FR for the evaluation of fatigue.

On the other hand, we aimed to study the effects of an isometric fatiguing hand task on peripheral and central motor excitability of control and MS participants (case-control study design).

Hence, we recorded 4 blocks of 40 trials each (20 MEPs and 20 CMAPs in a random order), one before the fatiguing task and 3 after it, as follows: baseline/T0 (before fatiguing task), T1 (immediately after the end of the task), T2 (1 min and 40 s after T1) and T3 (1 min and 40 s after T2). At the end of each session, all subjects were asked to report their level of perceived intensity during handgrip using the BRPES.

**Figure 1:**
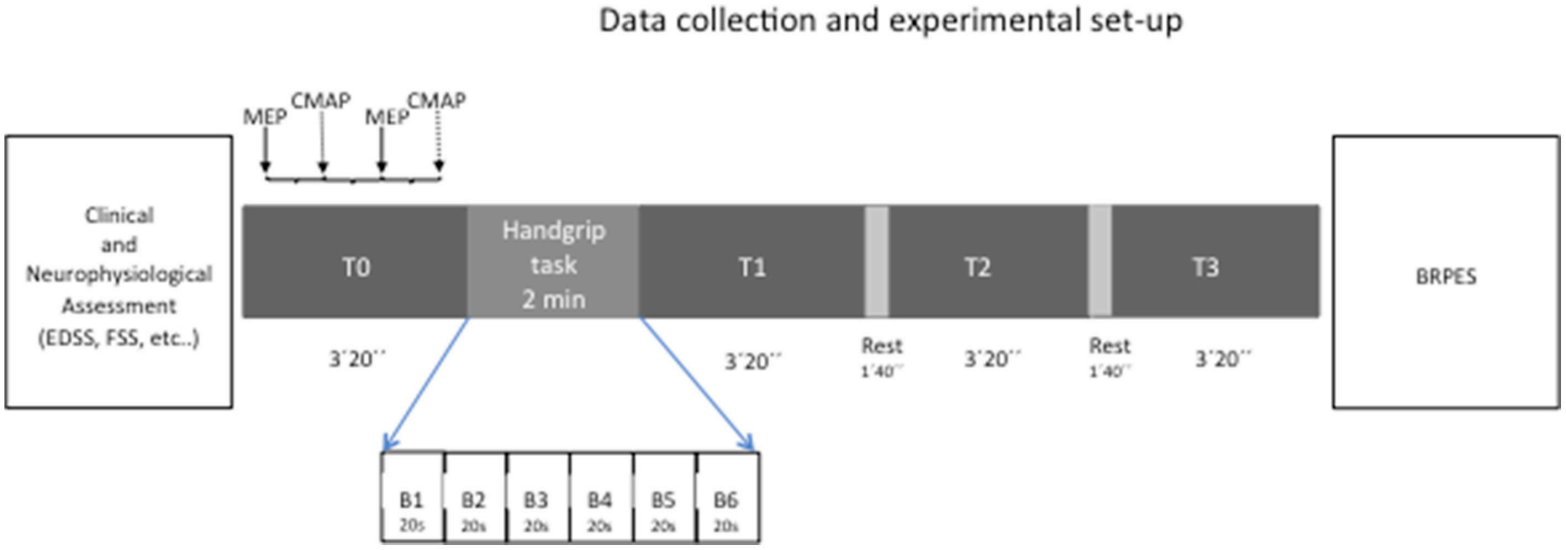
Schematic representation of the experimental setup.

In all sessions, subjects were seated comfortably and were instructed to refrain from speaking and to remain awake while in a calm, relaxed state till the beginning of handgrip task (and after the end of the handgrip task); the level of muscle contraction was continuously checked by means an audio-visual EMG feedback. The EMG background was measured in the 100 ms preceding both TMS and electric pulses to test the level of muscle relaxation along the experiment. Experimental design is represented in Figure 1.

## Statistical Analysis

Before entering ANOVA data were checked for normality by means of Kolmogorov-Smirnov test. Data presented are uncorrected for multiple comparisons.

### Healthy and MS Group Comparison.

Male/female ratio was compared using a χ^2^ test. FSS of both groups were compared using Mann-Whitney test. Moreover, a χ^2^ test was used to compare CSF of both groups. Mean age and FR of both groups were compared using *t*-test for unpaired data. As left hand is concerned, CMCT, PCT, RMT, eRMT, MEP_mod_, and CMAP_mod_, of both groups were compared by means of *t*-test for unpaired data. To disclose any difference between dominant and non-dominant side, right hand data (CMCT, PCT, RMT, eRMT, MEP_modulus_, and CMAP_modulus_) from MS group were compared with the MS group left hand data (Table 2) using unpaired *t*-test. As five patients were taking drugs to treat fatigue, we compared the FSS and FR of MS patients receiving anti-fatigue drugs with those not receiving.

**Table 2:** Summarized data of principle findings: comparison between healthy subjects and MS patients.

**Variable**	**Healthy subjects (left hand)**	**Multiple sclerosis (left hand)**	**p MS vs. HS (left hand)**	**Multiple sclerosis (right hand)**	**p MS left vs. right**
**CLINICAL VARIABLES**					
N	16	17			
EDSS		5.06 ± 1.93			
FSS	2.98 ± 0.91	4.67 ± 1.75	0.0014^***^	————–	————–
CSF (FSS≥4) n	3	11	0.0076^**^	————–	————–
FR	1.46 ± 1.22	4.92 ± 2.45	<0.0001^*^	————–	————–
**NEUROPHYSIOLOGICAL VARIABLES**					
N	16	16		17	
CMCT (ms)	4.6 ± 1.0	7.7 ± 4.1	0.0093^*^	8.7 ± 3.8	0.4798^*^
PCT (ms)	15.2 ± 0.7	15.4 ± 0.5	0.4295^*^	15.5 ± 0.7	0.6311^*^
RMT (% MSO)	44.9 ± 9.9	62.8 ± 24.7	0.0139^*^	68.8 ± 22.7	0.5239^*^
eRMT (mA)	12.46 ± 3.70	12.17 ± 3.34	0.8134^*^	12.25 ± 3.37	0.9143^*^
MEP (mV^*^s) 120%RMT	0.0129 ± 0.0070	0.0052 ± 0.0032	0.0007^*^	0.0077 ± 0.0063	0.1479^*^
CMAP (mV^*^s) 150%eRMT	0.0237 ± 0.0150	0.0141 ± 0.0030	0.0241^*^	0.0122 ± 0.0034	0.0999^*^
MF (T1/T0) (%)	72.916 ± 38.3	113.843 ± 51.1	0.0161^*^	98.102 ± 33.5	0.3299^*^
MF (T2/T0) (%)	84.856 ± 29.51	107.90 ± 40.42	0.0763^*^	105.493 ± 39.4	0.8685^*^
**MOTOR TASK AND PERCEIVED FATIGUE**					
N	16	16		17	
MHS (N)	239.485 ± 139.742	107.65 ± 43.898	0.0021^*^	125.253 ± 46.487	0.110^*^
FD (%)	73.562 ± 17.409	81.914 ± 29.48	0.3388^*^	80.216 ± 20.08	0.897^*^
BRPES	14.6 ± 1.7	13.7 ± 2.2	0.196^***^	13.6 ± 1.8	0.973^***^
POWER (N^*^s)	15492.26 ± 9832.35	5641.26 ± 3096.91	0.001^*^	7057.76 ± 3379.89	0.153^*^
POWER/BRPES	0.0013 ± 0.0008	0.013 ± 0.041	0.005^*^	0.0023 ± 0.001	0.222^*^

* , T test;

** , X^2^;

*** *, Mann-Whitney*.

*EDSS, Expanded Disability Status Scale; FSS, Fatigue Severity Scale; CSF, Clinically Significant Fatigue; FR, Fatigue Rating; CMCT, Central Motor Conduction Time; PCT, Peripheral Conduction Time; RMT, Resting Motor Threshold; eRMT, electrical Resting Motor Threshold; MEP, Motor Evoked Potential; CMAP, Compound Motor Action Potential; MF, Motor System Fatigability; MHS, maximal handgrip strength; FD, Force decay; BRPES, Borg Rating of Perceived Exertion Scale. Bold values indicate significant difference between groups*.

### Fatiguing Task

As left hand concerns, MHS and FD of both groups were compared using an unpaired *t*-test. Right hand data (MHS and FD) from MS group were compared with the MS group left hand data (Table 2) using unpaired *t*-test. BRPES were compared between MS patients and healthy subjects (and between hands in MS group) by means of Mann-Whitney test. FD was compared between groups (and in MS group between hands). Dynamometer signal over time was analyzed by dividing it in 6 20-sec-blocks: the modulus of each block was normalized to the MHS. Normalized data were entered into separate repeated-measures ANOVA, with BLOCK (B1, B2, B3, B4, B5, B6) as within-subject's factors and GROUP (MS, controls) as between-subject's factors. Fisher's Least significant difference (LSD) test was used for *post-hoc* comparisons.

### Effects of Fatiguing Task on Nervous System Excitability

Baseline MEP, CMAP and MF of both groups were compared using unpaired *t*-test. MEP and CMAP moduli were normalized to group-average baseline values before entering ANOVA. Note that this normalization, reduces between-group variability (imposing mean baseline values equal to 1), while maintaining the original within-group variability (scaling the variances), thus respecting the homeostadisticity assumption of the ANOVA (the variance in baseline is not zero after the normalization) (37). Afterwards, normalized values were entered into two separate repeated measures ANOVAs (GROUP x TIME) for MEP and CMAP. LSD significant difference test was used for *post-hoc* comparisons. Right hand data from MS group were compared with the MS group left hand data. All results were considered significant at *p* < 0.05.

In order to exclude the effects of changes in peripheral system, MEP data were normalized to the baseline Maximal M-wave (CMAP data) and these values were entered into two separate repeated measures ANOVAs (GROUP x TIME) for MEP and CMAP.

### Correlation Analysis

Both in patients and controls, we performed Spearman test between FSS and FR vs. maximal handgrip strength (MHS) and force decay (FD), between BRPES vs. FD and MF to explore the influence of fatigue trait on the handgrip task and the influence of fatigue state vs. force decay and the change in MEP_mod_ recorded after the task.

Only for MS patients, Spearman correlations between EDSS vs. FSS and FR and between FSS and FR vs. CMCT were performed to evaluate the impact of general fatigue on disability and the impact of corticospinal function on fatigue trait, respectively.

## Results

All the participants tolerated the whole experimental procedure and none experienced TMS related side effects. One MS participant had no recordable MEPs in the left FDI muscle and this side was excluded from final analysis of the neurophysiological variables. Kolmogorov-Smirnov test showed that data had normal distribution.

Data are summarized in Table 2.

### Healthy and MS Group Comparison: Baseline Condition

**Figure 2:**
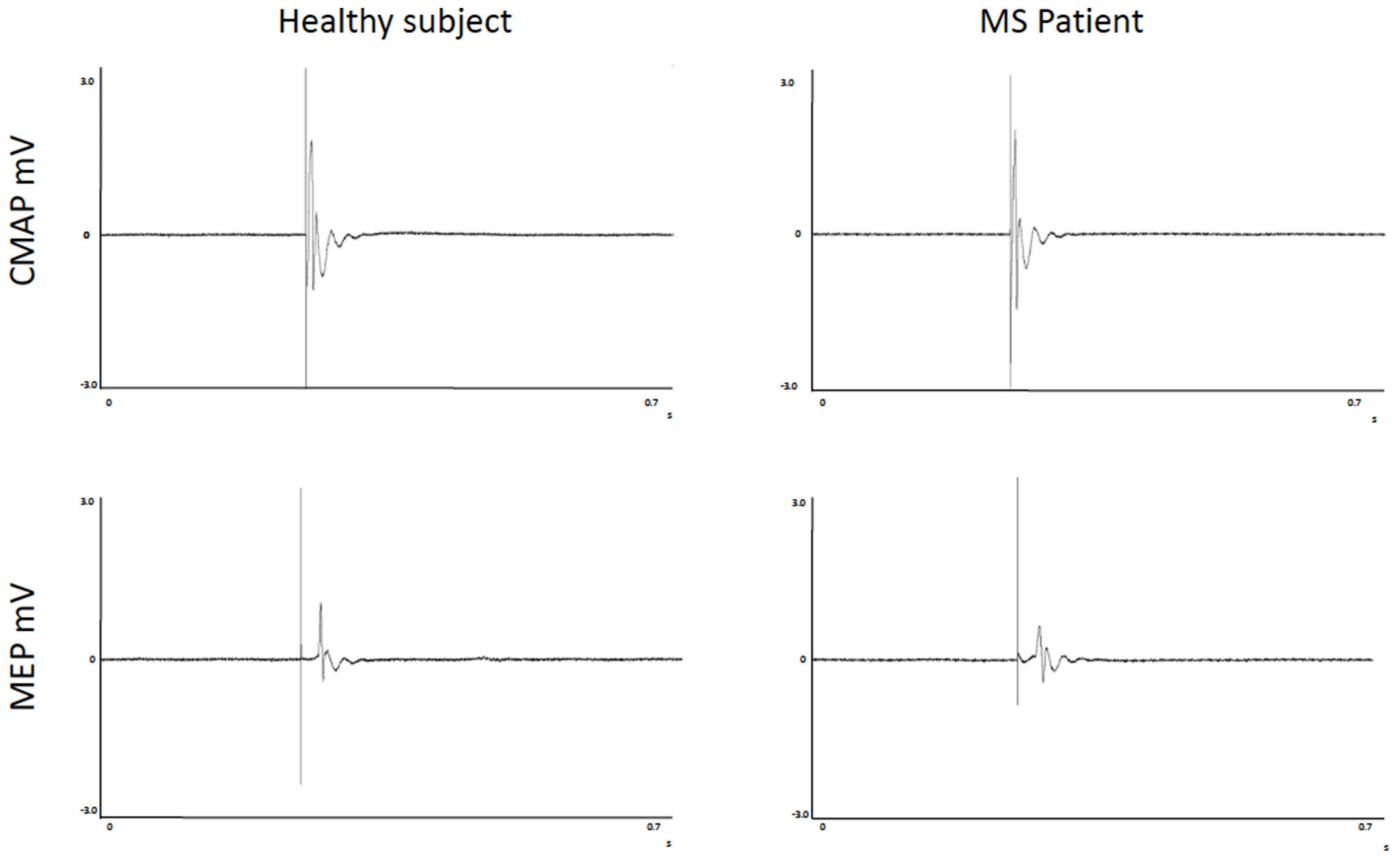
Recordings of Motor Evoked Potentials and Compound Motor Action Potentials in a representative control and a representative patient.

FSS scores, FR, CMCT, RMT, and MEP_mod_ were significantly different between groups (Table 2). Moreover, the CMAP_mod_ was smaller in MS group (Table 2). Clinically significant fatigue was present in 11/17 MS patients and in 3/16 of healthy subjects (*p* < 0.05). On the other hand, PCT, and eRMT were similar in both groups (Table 2). In MS group, no differences were observed between left and right hand (Table 2). No differences were observed between the MS patients taking and not taking anti-fatigue drugs in FSS, Fatigue Rating and Borg scale of both hands (all *p* > 0.5). Figure 2 shows single MEP and CMAP in a representative control and a representative patient.

### Fatiguing Task

**Figure 3:**
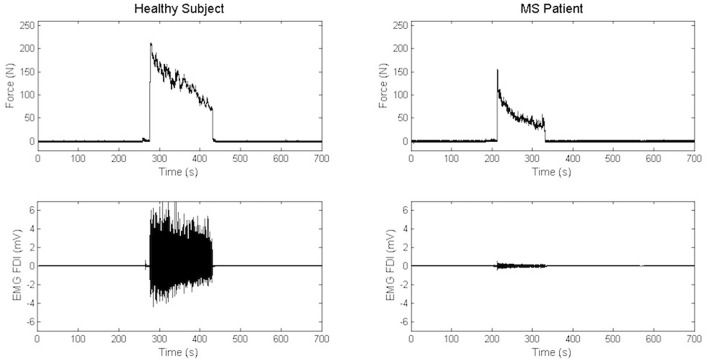
Recordings of electromyography background and handgrip task in a representative control and a representative patient.

Maximal Handgrip Strength was significantly lower in MS group than in the control group, while force decay and Borg Scale were similar in both groups. Recordings from a representative control and a representative patient are shown in Figure 3.

When comparing force decay and Borg Scale in dominant and non-dominant side in MS patients, no differences were found (Table 2). In few words, both patients and controls evaluated the muscular effort produced during handgrip as “somewhat hard.” Moreover, no differences were observed in Borg Scale when comparing left to right hand in patients (Table 2).

We compared force decay between groups (and in MS group between hands). Normalized data showed a progressive, significant decrement of the modulus over time [ANOVA, TIME: *F*_(5, 150)_ = 68.489, *p* < 0.001) in both groups without any significant difference between MS and control groups [ANOVA, TIME x GROUP: *F*_(5, 150)_ = 1.125, *p* = 0.35]. Similar results were obtained when left and right hand were compared in MS group [ANOVA, TIME: *F*_(5, 155)_ = 100.09, *p* < 0.001; TIME x HAND: *F*_(5, 155)_ = 1.35, *p* = 0.25].

### Effects of Fatiguing Task on Nervous System Excitability

**Figure 4:**
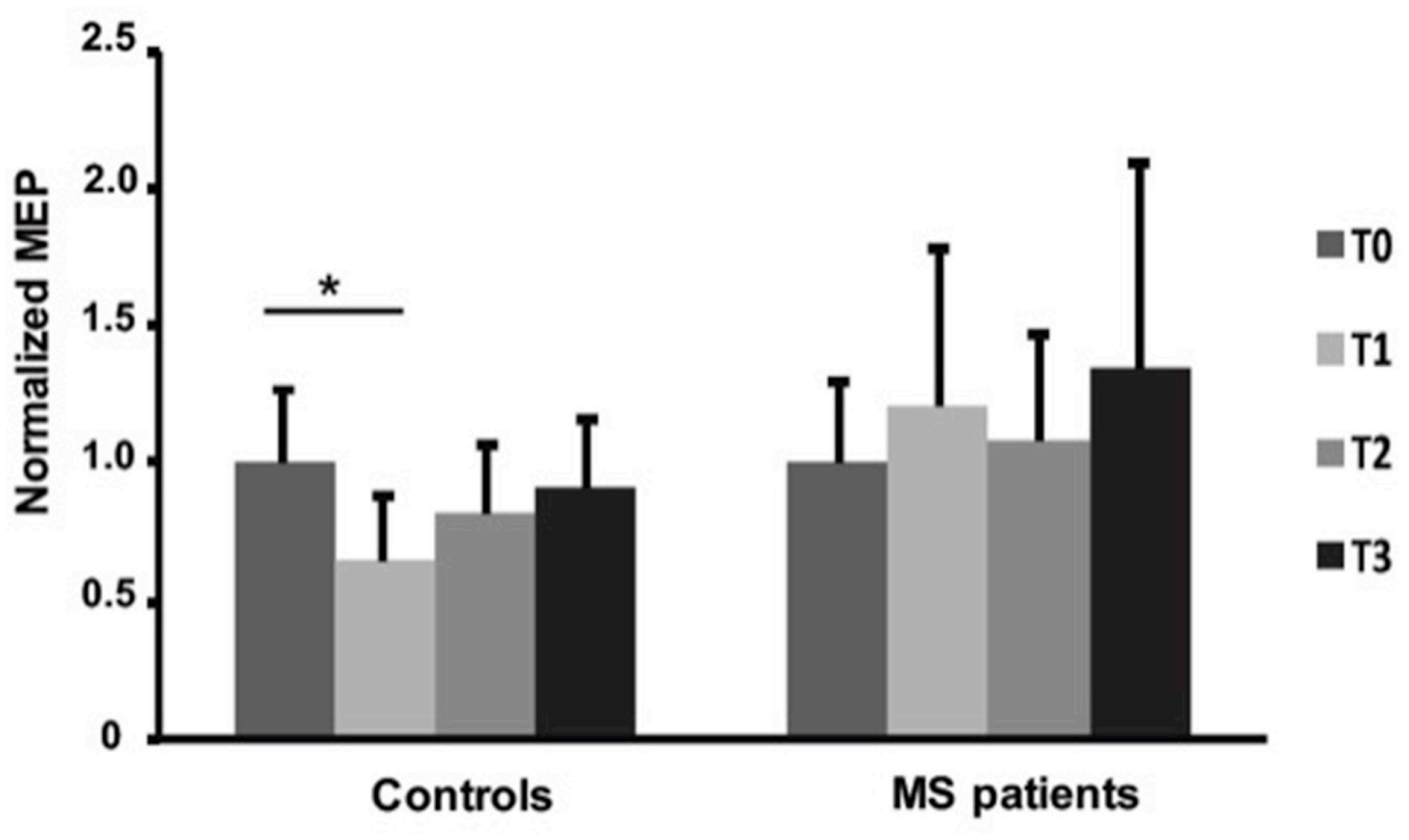
Normalized Motor Evoked Potentials in healthy subjects and MS patients at baseline and after fatiguing task. Error bars are standard deviations. *p < 0.05.

The effects of fatiguing task on normalized MEP_mod_ to baseline were significantly different between the two groups [ANOVA, TIME x GROUP: *F*_(3, 90)_ = 2.729, *p* = 0.048]. Particularly, normalized MEP_mod_ significantly decreased immediately after the fatiguing task (Fisher's LSD: T0 vs. T1; *p* = 0.0019) in the control group and then slightly recovered (Fisher's LSD: T0 vs. T2; *p* = 0.201 T0 vs. T3; *p* = 0.545). Particularly, immediately after the end of fatiguing task, MEP modulus was reduced with 27% in the control group while no changes were observed in the MS group over time (Figure 4). MF was different between MS and control groups (T1/T0, *p* = 0.0161; T2/T0, *p* = 0.0763] with bigger MF in controls than in patients (Table 2). No differences were observed between left and right hands in MS group (Table 2).

The effects of fatiguing task on normalized MEP_mod_ to baseline maximal M-wave were significantly different between the two groups [ANOVA, TIME x GROUP: *F* (_3, 90)_ = 5.123, *p* = 0.002]. Particularly, normalized MEP_mod_ significantly decreased after the fatiguing task (Fisher's LSD: T0 vs. T1, *p* < 0.001; T0 vs. T2, *p* = 0.018) in the control group and then slightly recovered (Fisher's LSD: T0 vs. T3, *p* = 0.268).

Furthermore, significant differences were observed after fatiguing task on the normalized CMAP_mod_ to baseline [ANOVA, TIME x GROUP: *F*_(3, 90)_ = 3.701, *p* = 0.015]. More in details, normalized CMAP_mod_ significantly decreased immediately after the fatiguing task (Fisher's LSD: T0 vs. T1; *p* = 0.0038 T0 vs. T2; *p* = 0.0005 T0 vs. T3; *p* = 0.0069] in the control group whereas no changes were observed in the MS group over time.

The effects of fatiguing task on normalized CMAP_mod_ to baseline maximal M-wave were significantly different between the two groups [ANOVA, TIME x GROUP: *F*_(3, 90)_ = 2.943, *p* = 0.037]. Particularly, normalized MEP_mod_ significantly decreased after the fatiguing task (Fisher's LSD: T0 vs. T1, *p* = 0.008; T0 vs. T2, *p* = 0.001; T0 vs. T3, *p* = 0.014) in the control group whereas no changes were found in the MS group over time.

The EMG background was similar in both groups over time [ANOVA: TIME: *F*_(3, 90)_ = 66.54, *p* < 0.001; TIME x GROUP: *F*_(3, 30)_ = 0.5692, *p* = 0.6367]. The EMG background registered over the FDI significantly increase in the first time point after the fatiguing task in both groups (Fisher's LSD: T0 vs. T1; *p* < 0.001 in healthy subjects and T0 vs. T1; *p* < 0.001 in MS patients).

### Correlation Analysis

Positive correlation was found between FSS and force decay (Spearman: rho = 0.544, *p* = 0.029), FR and FD (Spearman: rho = 0.828, *p* < 0.001), and a negative correlation was found between Borg scale and force decay (Spearman: rho = −0.604, *p* = 0.013) just in healthy subjects.

Correlation analysis results are summarized in Table 3.

**Table 3:** Correlational analysis: Evaluation of the influence of habitual fatigue level on the handgrip task and the influence of perceived task-related fatigue vs. FD and MF and the impact of general fatigue on disability as well as the impact of corticospinal function on general fatigue.

**Correlation**	**MS patients**	**Healthy subjects**
FSS and MHS	Left hand	Rho = 0.059, p = 0.827
	Rho = −0.017, *p* = 0.948	
	Right hand	
	Rho = −0.020 *p* = 0.937	
FR and MHS	Left hand	Rho = 0.273, p = 0.307
	Rho = 0.140, *p* = 0.606	
	Right hand	
	Rho = 0.153, *p* = 0.557	
FSS and FD	Left hand	**Rho** **=** **0.544, p** **=** **0.029**
	Rho = 0.054, *p* = 0.841	
	Right hand	
	Rho = −0.060, p = 0.819	
FR and FD	Left hand	**Rho** **=** **0.828, p < 0.001**
	Rho = 0.078, *p* = 0.774	
	Right hand	
	Rho = −0.124, p = 0.636	
BRPES and FD	Left hand	**Rho** **=** **-0.604, p** **=** **0.013**
	Rho = 0.317, *p* = 0.231	
	Right hand	
	Rho = −0.059, *p* = 0.823	
MF (T1/T0*100) and BRPES	Left hand	Rho = -0.377, p = 0.712
	Rho = 0.131, *p* = 0.628	
	Right hand	
	Rho = −0.362, *p* = 0.152	
MF (T2/T0*100) and BRPES	Left hand	Rho = -0.009, p = 0.973
	Rho = 0.399, *p* = 0.126	
	Right hand	
	Rho = −0.190, *p* = 0.464	
EDSS and FSS	Rho = 0.164, *p* = 0.528	
EDSS and FR	Rho = 0.219, *p* = 0.398	
FSS and CMCT	Left hand	
	Rho = 0.384, p = 0.142	
	Right hand	
	Rho = 0.297, *p* = 0.247	
FR and CMCT	Left hand	
	Rho = 0.225, *p* = 0.402	
	Right hand	
	Rho = 0.294, *p* = 0.252	
MHS and CMCT	Left hand	
	Rho = −0.26, *p* = 0.33;	
	Right hand	
	Rho = −0.37, *p* = 0.14	

*Spearman test was conducted for correlational analysis*.

*EDSS, Expanded Disability Status Scale; FSS, Fatigue Severity Scale; CSF, Clinically Significant Fatigue; FR, Fatigue Rating; CMCT, Central Motor Conduction Time; MF, Motor System Fatigability; MHS, maximal handgrip strength; FD, Force decay; BRPES, Borg Rating of Perceived Exertion Scale. Bold values indicate significant difference between groups*.

## Discussion

Our data confirmed the expected baseline differences between MS patients and controls: particularly we found a reduced CMAP and MEP modulus and an increased RMT in MS patients when compared to controls. Our data fits well with those of previous studies demonstrating an impairment of corticospinal tract in MS (35, 36, 38, 39). On the other hand, we found a reduction of baseline CMAP modulus in MS patients. Although MS affects CNS, the existence of a subclinical involvement of the peripheral nervous system, thought to be associated to MS or induced by muscular non-use or disability in general, was previously demonstrated (40). In this scenario, we cannot exclude that MEP modulus reduction depends on the subclinical impairment of peripheral nerve targeting FDI. On the other hand, the absence of an impairment of the latency excludes gross peripheral conduction abnormalities and severe axonal damage in the studied peripheral nerve (see also study limitations). Furthermore, the reduction of MEP modulus in MS patients could be drug-dependent since the patients included in the present study were taking several CNS active drugs, as shown in Table 1. However, when the pharmacological treatments of our patients was reviewed, it could be shown that 2 patients were taking clonazepam (a GABA-A agonist), 5 patients were taking baclofen (a GABA-B agonist), 2 patients were taking pregabaline, 1 patient gabapentine, 2 patients amantadine, 1 patient modafinil, 3 patients cannabidiol, and only 4 patients were taking anti-depressant drugs (1 Venlafaxine 1 Fluoxetine and 2 Escitalopram). In this context, just GABA-A agonist could affect motor threshold (i.e., increasing it) and MEP amplitude (i.e., reducing it) whereas the other mentioned drugs did not substantially reduce motor cortex excitability as reported by Ziemann (41). When we take into account these observations we can suppose that the reduction of motor cortex excitability in the MS patients group was not primarily due to a pharmacological effect.

MS patients showed clinically significant fatigue in 2/3 of the patients (11/17), even considering that a few patients were receiving anti-fatigue drugs. Only four patients were taking anti-depressant drugs and none of the patients or subjects referred significant mood disorders during baseline clinical interview. It is demonstrated that mood as well as sleep disorders are common in MS and could participate in the perception of fatigue in daily live activities (42). We did not formally screened for sleep disorders, which in any case were not reported by the participants in the general interview.

As expected, patients presented diminished maximal handgrip strength and corticospinal dysfunction (prolonged CMCT). Moreover, there was a tendency to negative correlation between these latter parameters (the longer the CMCT the less the maximal handgrip strength). On the other hand, fatigue state shown by the motor decay that subjects experienced during the isometric fatiguing task was similar in both MS patients and healthy controls (of course MS patients started the task with much less strength). Both patients and controls had also similar perceived fatigue state of the executed task, but whilst in controls there was a correlation between the force decay and both for general fatigue trait (scores FSS and FR) and fatigue state (Borg scale), in the MS group this correlation was not present. In other words, fatigue trait and fatigue state in MS patients are independent from handgrip strength as well as fatigue state is independent from fatigue trait. Moreover, FSS and force decay were independent from the corticospinal tract dysfunction (CMCT). As FSS concerns, we have to take into account that is a multidimensional scale used for a gross evaluation of fatigue in daily life, so its relationship with the corticospinal tract can be tiny. Furthermore, lack of correlation between corticospinal tract dysfunction and force decay suggests that force decay induced by an isometric task does not depend on corticospinal dysfunction in MS. Differently from control group, we were unable to detect clear MF (by TMS after the handgrip task) or a clear reduction in peripheral nerve excitability in MS. Previous studies reported a lower decay in cortical excitability (43) or an increase (i.e., compensatory) in central motor drive in MS patients (23, 44) even if these authors reported also a similar reduction of peripheral nerve excitability in MS and controls. This discrepancy was probably due to the normality of CMCT in MS patients found in previous studies (43), while patients included in our study were characterized by CMCT alteration. This suggests that in our MS patients, the dysfunction in corticospinal connection (i.e., increased CMCT), reduced the torque realized during the task (MHS lower than controls), impeded, together with the subclinical alteration in peripheral nervous system demonstrated in patients, a reduction in peripheral nerve excitability and induced a compensatory increase in motor cortex excitability, that prevented MEP reduction after fatiguing task.

Following correction, taking in consideration the real total effort performed by the subjects (THS/BRPES), we can note that the perceived fatigue is much more in the MS group. Based on these data, a number of considerations could be suggested with proper caution: (1) Fatigue trait does not depend on corticospinal dysfunction in MS; (2) force decay induced by an isometric task does not depend on corticospinal dysfunction in MS; (3) maximal handgrip strength depends on corticospinal dysfunction in MS; (4) Perceived fatigue in MS is higher than in controls (similar perceived fatigue with much less THS); (5) MEP and CMAP changes after 2-min isometric fatiguing task are not able to measure fatigue in MS, probably due to a central motor drive compensation.

## Central Fatigue Generation

MEP amplitude indirectly measures the descending corticospinal drive and, in healthy subjects, fatigue affects this descending activity (32).

In our experiment, healthy subjects showed a reduction in MEP modulus in line with other previous results that report an increase during the fatiguing task and a post-fatigue reduction in MEP amplitude (17). However, as elegantly described by Gandevia, MEP amplitude increased after 2 min of MVC just for the first 30 s and after 60 s began to decrease (20), so it is not surprising that our data demonstrated a MEP reduction in healthy subjects in the first minutes recording block as facilitation prevails just for the first 30 s. The post-exercise facilitation is probably due to the difficulty to keep the muscle fully relaxed after the fatiguing task (20). Our data on EMG background confirm the reduced relaxation immediately after the task. On the other hand, in the first block after the fatiguing task we observed similar values of the EMG background for controls and MS subjects, suggesting that the difference in MEP changes could not be explained by different muscle relaxation capacity between the two groups.

This point could further suggest that controls show a reduction in corticospinal output, after fatiguing task, while MS patients do not. In this context, it could be considered that fatigue state in MS patients also depends on connectivity changes in motor and non-motor basal ganglia, including motivation and reward circuits (13, 45, 46) and could be also due to adaptive changes that take place in MS pathological process along with the whole duration of the illness, related to clinical and subclinical neuropsychiatric changes, as well as depression and cognitive impairment (47, 48). Furthermore, fatigue-induced cortical hyperexcitability was already described in MS patients (23, 44). The two phenomena could indicate a cortical reorganization that might serve to compensate for progressive tissue damage by MS (49) and could contribute to fatigue pathophysiology in multiple sclerosis (i.e., the more the reorganization the more central fatigue).

## Limitations of the Study

It is important to note that the present study presents some limitations that could affect result interpretation.

We recruited the MS study participants in a consecutive manner from a patient association, and for this reason the cohort of the MS group was not homogeneous. Patients were taking different disease-modifying drugs and symptomatic medications that may affect our findings. Unfortunately, due to the limited number of patients included in the study, we could not perform further analysis to differentiate the effects of each drug on our results. For example, it should be considered that interferon could affect fatigue itself, directly but also indirectly, by means of a drug-induced worsening of sleep duration, quality, and stability (50). Moreover, we did not formally screen patients for sleep disorders, so that we cannot quantify and evaluate the sleep-induced effects on fatigue.

Another limitation is the lack of further neurophysiological work-up in our study: particularly we did not evaluate other TMS protocols that can provide information about GABA-A and GABA-B circuits or plasticity. However, our main objective was to observe and study the changes in fatigue induced by a handgrip and its relationship with corticospinal tract, so we considered that paired-pulse TMS and protocols to evaluate plasticity were beyond the main objective of the present study.

Furthermore, we did not analyze the neuroradiological findings at the moment of the study (or close by the study). Similarly, we do not have a formal clinical neurophysiological assessment of the peripheral nervous system (motor and sensory nerve conduction studies, F-waves, H-reflexes, and needle EMG). Obviously, the lack of these kinds of information prevented the execution of further correlations' analysis that could have been provided further relevant details about the phenomena observed.

## Conclusions

Since MS patients and healthy subjects present important neurophysiological differences in baseline conditions, also fatigue generation mechanisms were different between the two groups. In healthy subjects, isometric fatiguing task induces a central fatigue that involves corticospinal functions as demonstrated by MEP reduction and confirmed by analyzing data normalized to M-wave excluding by this way a possible effect over the peripheral nervous system. In MS patients, the pathogenesis of fatigue seems not driven by the mechanisms directly related to corticospinal function (that characterize fatigue in controls) but seems probably due to other “central abnormalities” upstream to primary motor cortex (11).

## Ethics Statement

This study was carried out in accordance with the recommendations of Toledo Area Ethical Committee. The protocol was approved by the Toledo Area Ethical Committee. All subjects gave written informed consent in accordance with the Declaration of Helsinki.

## Author Contributions

LM-M, NM-L, MD, MR, JA, and AO conceived of the study and designed the experimental paradigm. VS-L, MT-P, DP-P, and CA-B performed the experiments and analyzed the data. LM-M and AO wrote the manuscript. LM-M, NM-L, MD, MR, and JA provided feedback and edited the manuscript. All authors read and approved the final manuscript.

### Conflict of Interest Statement

The authors declare that the research was conducted in the absence of any commercial or financial relationships that could be construed as a potential conflict of interest.
